# Author Correction: Multiple antiferromagnetic phases and magnetic anisotropy in exfoliated CrBr_3_ multilayers

**DOI:** 10.1038/s41467-023-41613-y

**Published:** 2023-09-18

**Authors:** Fengrui Yao, Volodymyr Multian, Zhe Wang, Nicolas Ubrig, Jérémie Teyssier, Fan Wu, Enrico Giannini, Marco Gibertini, Ignacio Gutiérrez-Lezama, Alberto F. Morpurgo

**Affiliations:** 1 Department of Quantum Matter Physics, University of Geneva, 24 Quai Ernest Ansermet, CH-1211 Geneva, Switzerland; 2 Department of Applied Physics, University of Geneva, 24 Quai Ernest Ansermet, CH-1211 Geneva, Switzerland; 3 Advanced Materials Nonlinear Optical Diagnostics lab, Institute of Physics, NAS of Ukraine, 46 Nauky pr., 03028 Kyiv, Ukraine; 4 MOE Key Laboratory for Nonequilibrium Synthesis and Modulation of Condensed Matter, Shaanxi Province Key Laboratory of Advanced Materials and Mesoscopic Physics, School of Physics, Xi’an Jiaotong University, Xi’an, 710049 China; 5 Dipartimento di Scienze Fisiche, Informatiche e Matematiche, University of Modena and Reggio Emilia, IT-41125 Modena, Italy; 6 Centro S3, CNR-Istituto Nanoscienze, IT-41125 Modena, Italy

**Keywords:** Magnetic properties and materials, Two-dimensional materials

Correction to: *Nature Communications* 10.1038/s41467-023-40723-x, published online 17 August 2023

The acknowledgments of this article did not include the project number of the Swiss National Science Foundation (Division II, project #200020_178891). This has been corrected in the main text.

