# Supplementary material for: Multiple antiferromagnetic phases and magnetic anisotropy in exfoliated CrBr3 multilayers
Source: Nat Commun. 2023 Aug 17;14:4969. doi: 10.1038/s41467-023-40723-x (PMC10435511; doi:10.1038/s41467-023-40723-x)
Supplement: Supplementary file 1 — Supplementary Information [file 41467_2023_40723_MOESM1_ESM.pdf]

Supplementary Information for

Multiple antiferromagnetic phases and magnetic anisotropy in exfoliated $CrBr_3$ multilayers

Fengrui Yao[1,2*], Volodymyr Multian[1,2,3], Zhe Wang[4*], Nicolas Ubrig[1,2], Jérémie Teyssier[1,2], Fan Wu[1,2], Enrico Giannini[1], Marco Gibertini[5,6], Ignacio Gutiérrez-Lezama[1,2] and Alberto F. Morpurgo[1,2*]

[1] *Department of Quantum Matter Physics, University of Geneva, 24 Quai Ernest Ansermet, CH-1211 Geneva, Switzerland*

[2] *Department of Applied Physics, University of Geneva, 24 Quai Ernest Ansermet, CH-1211 Geneva, Switzerland*

[3] *Advanced Materials Nonlinear Optical Diagnostics lab, Institute of Physics, NAS of Ukraine, 46 Nauky pr., 03028 Kyiv, Ukraine*

[4] *MOE Key Laboratory for Nonequilibrium Synthesis and Modulation of Condensed Matter, Shaanxi Province Key Laboratory of Advanced Materials and Mesoscopic Physics, School of Physics, Xi'an Jiaotong University, Xi'an, 710049, China*

[5] *Dipartimento di Scienze Fisiche, Informatiche e Matematiche, University of Modena and Reggio Emilia, IT-41125, Modena, Italy*

[6] *Centro S3, CNR-Istituto Nanoscienze, IT-41125, Modena, Italy*

*Correspondence: fengrui.yao@unige.ch; zhe.wang@xjtu.edu.cn; alberto.morpurgo@unige.ch

Further characterization of the $CrBr_3$ crystals and of the tunnel barriers used in our work

In the main text, we conclude that exfoliated $CrBr_3$ multilayers are found in three locally stable crystal structures, referred to as AA, M and AB stacking, and infer from Raman and tunneling magnetoconductance measurements that the interlayer magnetic coupling is ferromagnetic in AB stacking and antiferromagnetic in AA and M stacking, as predicted by the DFT calculations of Ref.[1]. $CrBr_3$ multilayers exhibiting long sequences of AB-stacked layers give rise to the magnetoconductance response characteristic of the ferromagnetic phase widely discussed in the literature[2-4]. For AA- and M-stacked multilayers giving rise to antiferromagnetism, our work shows that long sequences occur with different frequencies and that a single tunnel barrier commonly contains sequences of layers exhibiting different structures. Here we show additional data to substantiate different detailed aspects of the claims that we make in the main text.

In Supplementary Fig. 1, we present Raman spectra of two representative bulk crystals extracted from the same growth batch, which we use to determine that parts with different structures are present. Specifically, we find that one of the crystals only exhibits the Raman peaks expected for the thermodynamically stable structure of $CrBr_3$. In the other, however, an additional peak is present at 161 cm^{-1}, which we take as evidence for the presence in the crystal of a large amount of a different $CrBr_3$ allotrope (i.e., a phase in which the $CrBr_3$ planes are stacked differently than in the thermodynamically stable phase). Realizing devices based on multilayers exfoliated from crystals showing a peak at 161 cm^{-1} with large intensity makes the presence of antiferromagnetic inclusions in the barrier more likely.

In Supplementary Fig. 2, we show data from all measured tunnel barriers in which magnetoconductance jumps have been detected, which illustrate how in different devices contributions attributed to the two antiferromagnetic phases and to the ferromagnetic one are present with different magnitude.

In Supplementary Fig. 3 we show Raman spectra taken at three positions of two tunnel barrier devices realized on thin $CrBr_3$ multilayers, to illustrate the uniformity of the signal and hence the uniformity of the stacking.

In Supplementary Fig. 4 we show the magnetoconductance of the four-layer tunnel barrier discussed in the main text, in which all layers are M-stacked (L-type antiferromagnetic interlayer coupling), for opposite polarities of applied bias. In the Fowler-Nordheim regime the tunneling magnetoconductance is more sensitive to the layers close to the contact injecting electrons, and reversing the bias corresponds to injecting the tunneling electrons from either the top or the bottom electrode (see Supplementary Fig. 4d). Comparing the magnetoconductance measured for opposite polarities therefore allows us to determine whether the multilayer has or not the same structure on both sides.

Finally, in Supplementary Fig. 5, we show data with opposite polarity of applied bias for the AA-stacked junction presented in the Fig.5d of the main text. In the main text, we state that in that junction two layers near the injecting contact exhibit S-type stacking and that in the rest of the junction the layer stacking corresponds to interlayer ferromagnetic coupling, as it can be evinced from the full temperature and magnetic field dependence of the magnetoconductance. To substantiate these statements in more detail, in Supplementary Fig. 5 we show the temperature and magnetic field dependence of the magnetoconductance measured for opposite polarities.

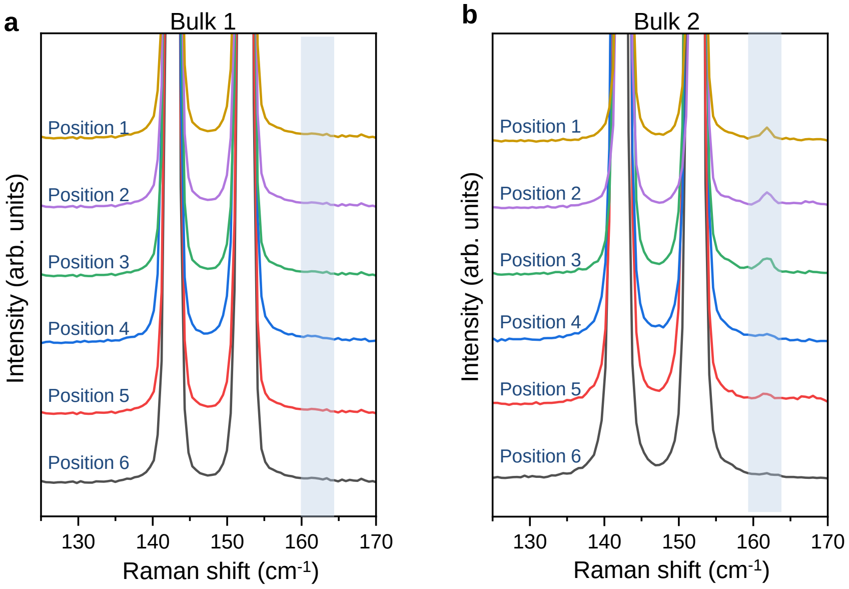

Supplementary Fig. 1. Raman spectroscopy performed at different positions of two different bulk crystals at T = 10 K. a, Raman spectroscopy measurements performed on different positions of a same crystal show exclusively the peaks expected from the thermodynamically stable (ferromagnetic) structural phase. **b**, Crystals exhibiting an additional Raman peak at approximately 161 cm^{-1} (blue shadow) can be found in all growth batches, as well as in crystals purchased from commercial providers. We attribute this additional peak to the presence of a considerable amount of a second allotrope of $CrBr_3$ with a different layer stacking. This conclusion is fully consistent with transmission electron microscopy data reported in the literature[5].

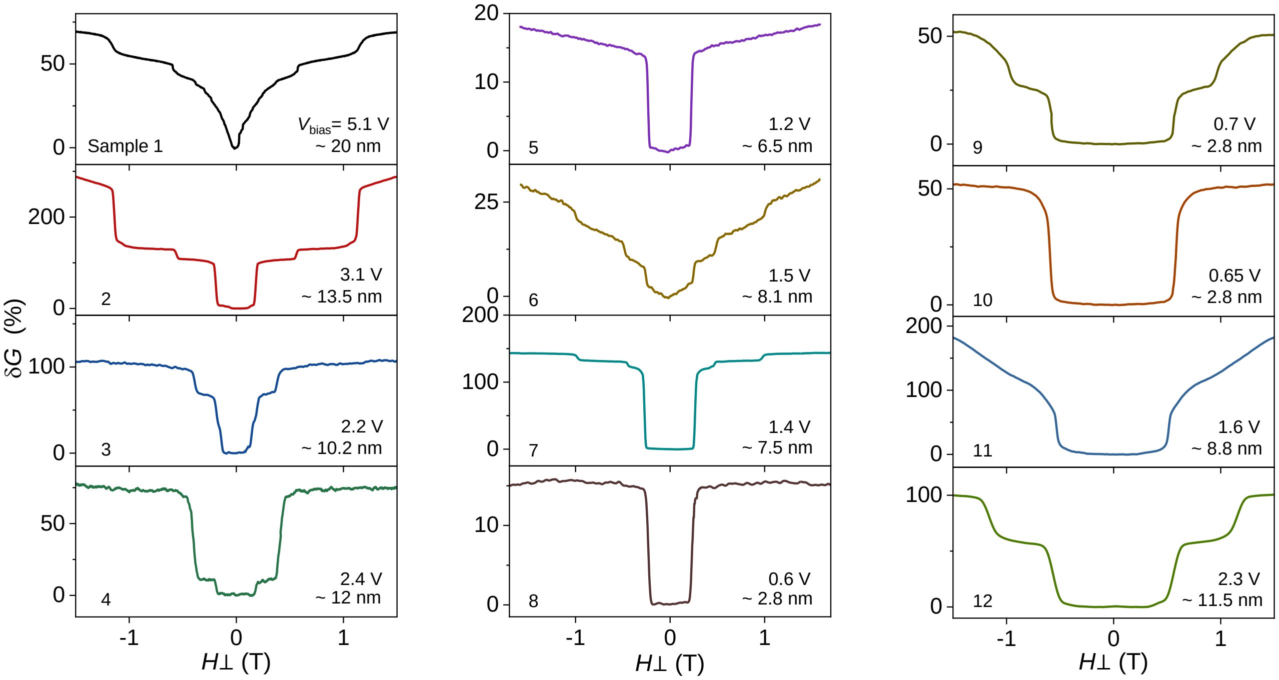

Supplementary Fig. 2. Low- temperature (*T* = 2K) tunneling magnetoconductance (δ*G*) of 12 $CrBr_3$ multilayers exhibiting antiferromagnetic coupling. As discussed in the main text, the presence of jumps in the magnetoconductance allows the presence of two different antiferromagnetic phases to be identified, and provides information about the number of layers stacked in the corresponding antiferromagnetic phase. For completeness, here we show magnetoconductance traces for all devices that we measured in which we observed antiferromagnetic interlayer coupling (the sample numbers shown in each panel correspond to the sample numbers in Fig. 3c). The data clearly show that different situations can occur, in which either one or the other type of antiferromagnetism – or both –are detected in the magnetoconductance. In addition, a smooth magnetoconductance background can also be present in some devices, due to the concomitant presence of layers with ferromagnetic interlayer coupling (the $CrBr_3$ multilayers thickness varies from 2.8 to 20 nm. The applied voltage –indicated in the corresponding panel– is increased for thicker devices, to ensure that the tunnelling current is large enough to be measured. For a given voltage polarity, changing the magnitude of the voltage bias does not affect the general behaviour of magnetoconductance, and only changes its magnitude.

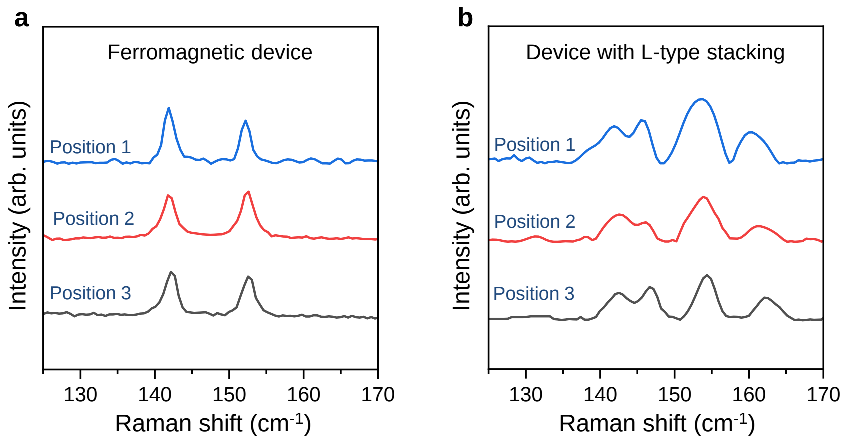

Supplementary Fig. 3. Homogeneity of Raman spectra on tunnel barrier devices. Raman spectra measured at three different positions of a device showing (a) ferromagnetic behaviour and (b) L-type antiferromagnetic behavior.

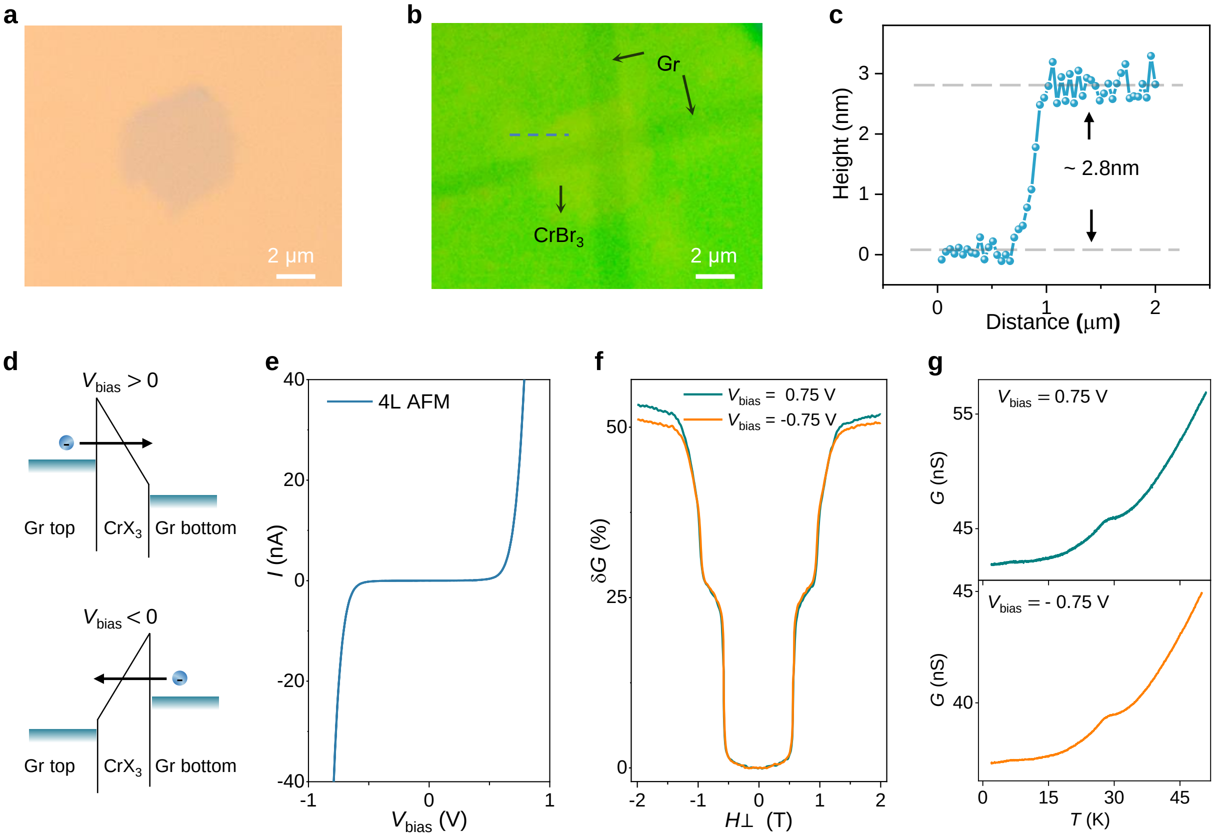

Supplementary Fig. 4. Transport measurements on a L-type 4L antiferromagnetic $CrBr_3$ tunnel barrier with different bias polarity. a, Optical microscope image of the 4L $CrBr_3$ crystal used as the barrier, as exfoliated onto a 285 nm SiO_2/Si substrate. **b**, Optical microscope image of the tunnel junction after assembly of the van de Waals heterostructure (h-BN/graphite (Gr) /$CrBr_3$/graphite/h-BN). **c**, Atomic force microscope height profile of the $CrBr_3$ crystal measured along the dotted line in panel (b). The 2.8 nm step height corresponds to 4 layers (4L). **d**, Energy band diagram of the tunnel junctions in the Fowler-Nordheim (FN) tunneling regime, illustrating schematically why the polarity of the applied bias (V_{bias}) determines the sensitivity of transport to the layers closer to the injecting contact. **e,** Low-temperature ($T = 2$ K) tunneling current (I) across the $CrBr_3$ tunnel barrier as a function of V_{bias} at zero magnetic field, exhibiting nearly symmetric response for opposite bias polarities. **f**, The magnetoconductance $\delta G(H, 2\text{K})$ measured for positive (green) and negative (orange) voltage bias, virtually coincides, showing that the layers near opposite electrodes have the same (L-type) stacking. **g,** The temperature dependence of the tunneling conductance G (at H = 0 T) also shows virtually identical behavior for both polarities, with an enhanced conductance suppression below T_N (kink in the curves), as expected for antiferromagnetic ordering[6] (see main text).

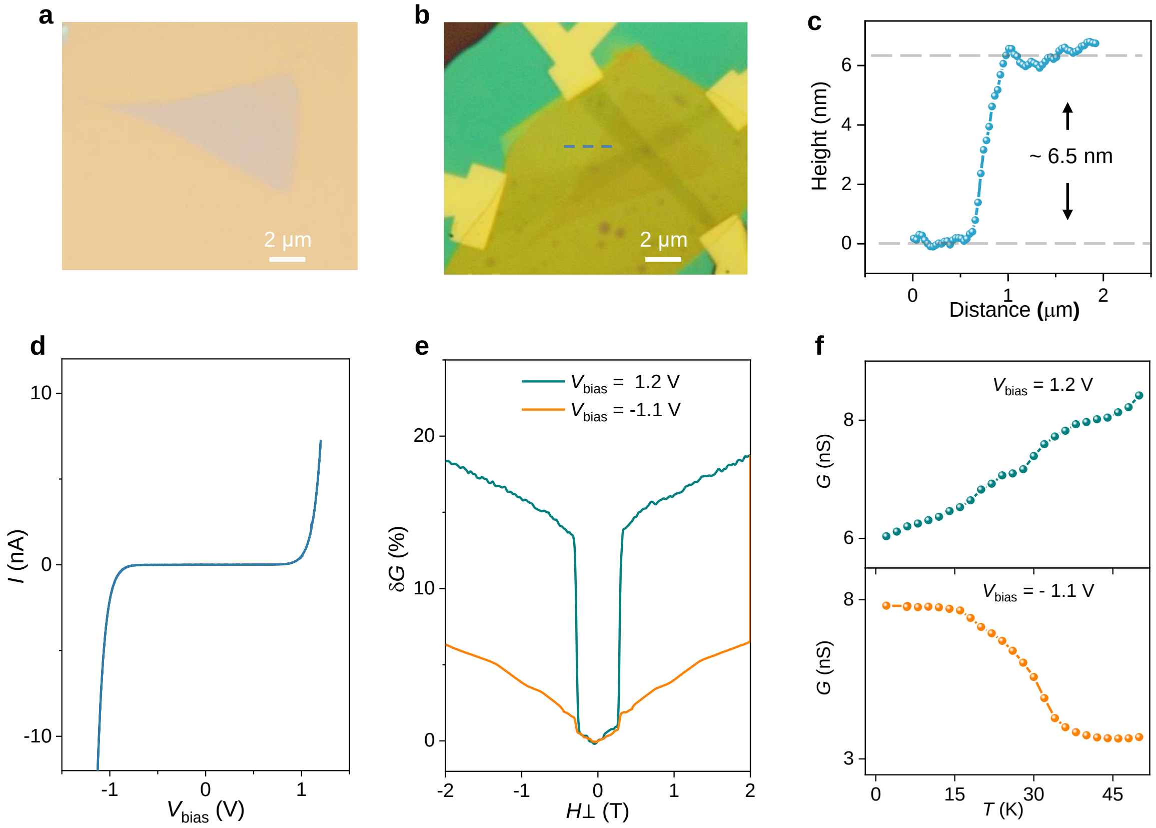

Supplementary Fig. 5. Characterization of the S-type stacked $CrBr_3$ tunnel barrier used in the main text to determine T_N for the S-type antiferromagnetic phase. a, Optical microscope image of the $CrBr_3$ crystal used as a barrier, as exfoliated on a 285 nm SiO_2/Si substrate. **b**, Optical microscope image of the complete tunnel junction device based on a h-BN /graphite/$CrBr_3$/graphite/ h-BN van de Waals heterostructure. **c**, Atomic force microscope height profile of the $CrBr_3$ flake measured along the dotted line in panel (b), showing a step height of ~ 6.5 nm. **d**, The low-temperature (T = 2 K) tunneling *I-V* characteristics of the $CrBr_3$ tunnel barrier exhibit a clear asymmetry. **e**, Magnetoconductance $\delta G(H, 2\text{K})$ measured for positive (green) and negative (orange) voltage bias. For positive bias a single jump at 0. 2T is observed, indicating that two AA-stacked layers are present close to the top electrode; for negative bias the magnetoconductance is typical of a ferromagnetic barrier, indicating that the layers next to the bottom contact are AB-stacked. **f,** Temperature dependence of the tunneling conductance G (at H = 0 T) exhibits opposite trends for opposite polarities, consistently with the effect of S-type antiferromagnetically coupled layers for positive polarity (green curve) and of ferromagnetically coupled layers for negative polarity (orange curve; see discussion in the main text).

Reference:

1 Gibertini, M. Magnetism and stability of all primitive stacking patterns in bilayer chromium trihalides. *J Phys D* **54,** 064002 (2020).

2 Ghazaryan, D. et al. Magnon-assisted tunnelling in van der Waals heterostructures based on $CrBr_3$. *Nat Electron* **1,** 344-349 (2018).

3 Kim, H. H. et al. Evolution of interlayer and intralayer magnetism in three atomically thin chromium trihalides. *PNAS* **116,** 11131-11136 (2019).

4 Wang, Z. et al. Magnetization dependent tunneling conductance of ferromagnetic barriers. *Nat Commun* **12,** 6659 (2021).

5 Han, X. et al. Atomically unveiling an atlas of polytypes in transition-metal trihalides. *J. Am. Chem. Soc.* **145,** 3624-3635 (2023).

6 Wang, Z. et al. Very large tunneling magnetoresistance in layered magnetic semiconductor CrI_3. *Nat Commun* **9,** 2516 (2018).
